# An intelligent fuzzy-particle swarm optimization supervisory-based control of robot manipulator for industrial welding applications

**DOI:** 10.1038/s41598-023-35189-2

**Published:** 2023-05-22

**Authors:** A. Sathish Kumar, S. Naveen, R. Vijayakumar, V. Suresh, Abdul Rab Asary, S. Madhu, Kumaran Palani

**Affiliations:** 1 Department of Electrical and Electronics Engineering, Holy Mary Institute of Technology and Science, Hyderabad, India; 2 Department of Automobile Engineering, Saveetha School of Engineering, Saveetha Institute of Medical and Technical Sciences, Chennai, India; 3 Department of Electrical and Electronics Engineering, Christ Institute of Technology, Puducherry, India; 4 Department of Mechanical Engineering, Adhi College of Engineering and Technology, Kanchipuram, India; 5 University of Naples, Parthenope, Italy; 6 Department of Mechanical Engineering, College of Engineering, Wolaita Sodo University, Wolaita Sodo, Ethiopia

**Keywords:** Engineering, Mathematics and computing

## Abstract

The propensity of manufacturers to produce goods at affordable cost, with more accuracy, and at a faster rate force them to search for novel solutions, such as deploying robots in place of people in a sector that can accommodate their needs. Welding is one of the most crucial processes in the automotive industry. This process is time-consuming, subject to error, and demands skilled professionals. The robotic application can improve this area of production and quality. Other industries, such as painting and material handling, can also profit from the use of robots. This work describes the fuzzy DC linear servo controller, which functions as a robotic arm actuator. Robots have been widely employed in most productive sectors in recent years, including assembly plates, welding, tasks at higher temperatures, etc. Controlling a robot accurately is a difficult undertaking as a robot is very nonlinear with many joints that are often organized and unstructured. To carry out the effective task, an effective PID control based on fuzzy logic has been employed together with the method of Particle Swarm Optimization (PSO) approach for the estimate of the parameter. This offline technique determines the lowest number of optimal robotic arm control parameters. To verify the controller design with computer simulation, a comparative assessment of controllers is given by means of a fuzzy surveillance controller with PSO which improves the parameter gain to provide a rapid climb, a smaller overflow, no steady condition error signal, and effective torque control of the robot arm.

## Introduction

Robotic technology is now being employed in numerous sectors to improve production precision and excellence. Before robots are commissioned in the industries, controlled application and control parameters have to be addressed in the significant measure since robot handling is extremely nonlinear in structured and unstructured settings, with varied uncertainties. Therefore, it is highly necessary for parametric estimation and analysis before utilizing it in any real-time activity. Mathematical modeling is suitable for organized settings and is followed by any robotic control. On the other hand, because of the many uncertainties that are present in their joints, their operation in unstructured settings would result in extra insecurities and thus must be taken into consideration properly, particularly while developing their model. Usually, these uncertainties arise because of a lack of sensor accuracy and inaccurate environmental forecast. To solve the above-mentioned challenges, an improved hybrid control system was suggested that knows the precise robot location and strength control. The precise placement and control need huge parameters and more complicated offline analysis. A neural controller was described for effective management of the uncertainty caused by effective parameters owing to joint occurrences and environmental features and robotic dynamics for effective positioning, forcing, and joint subspace^1–4^. As the efficacy of the neuro-controller depends on more and more training patterns with a vast array of uncertainties, it cannot give improved outcomes and is thus less appropriate in real-time operation. Supported by the stability of Lyaponove, an adaptive robust hybrid position and force, control approach for robotic manipulator estimate was suggested^5,6^. No information relating to uncertainty is required for this controller. It is very difficult to maintain accuracy again and over again without this information. The algebraic approach for reverse cinematic operations was found to have been used, which does not guarantee closed solutions. In addition, it may be done with iterative approaches, but converges into a single answer only and will not work almost individually.

In Refs.^7,8^ a hybrid position/force control was introduced, which is a stable widespread design, and may affect both torque and joint position. Due to the inverse of the Jacobian matrix manipulator, the system may have movie instability. A fuzzy controller is very useful in the domains of lots and many uncertainties and unexpected settings, and also has great capabilities for solving issues in the real world through the right design of robotic controllers. Because of its non-linear properties, this fuzzy controller is guaranteed to be superior compared to the PID. A mix of conventional and flexible procedures is developed and provided to effectively construct and get the robotic handler that tackles the difficulties of similarity between regular and Fuzzy logic methods. Depending on the input variables, the fuzzy logical control system has been designed with two inputs to identify the selection of input variables, which is a more single task^9^. The new fuzzy input PD and PI control error change and error control signals, with similar input variables by type PI, position, and speed controller type^10–13^. These two controllers have their advantages and restrictions, e.g. PD can eliminate permanent state faults, while PI does have a limitation to improving transient response. Late multiple input or partially fluid modified/based linear propositional, integral, and derivative controls were used to correct all the disadvantages in the inverse movie system. In robot manipulators, the PSO method improves the optimization control utilized to regulate the DC motor parameter^14^. The sophisticated technique PSO mutation algorithm is utilized to address the industrial robot handler optimization problem^15,16^. The reverse dynamic control of the robot manipulator using fuzzy and PSO controllers is presented for checking the optimum problem^17,18^.

This study examines the finding of a robust technique for the active robot arm process by turning using the innovative monitoring control Fuzzy-PSO, wherein the Fuzzy and the PSO are used to detect the various characteristics of the DC servo motor. This engine typically informs the robotic arm manipulator about the degree of freedom of different input–output parameters derived with the aid of extracting features. The fuzzy logic describes the fuzzy sets of membership functions and the rule of inference mechanism to detect system relationships for input and output. To recapitulate, the study begins with a discussion of the robot and its dynamics before explaining its structure and the method of extracting parameters through the inclusion of the PSO, followed by an inquiry on the fugitive controller.

## Robotic systems and dynamics

**Figure 1:**
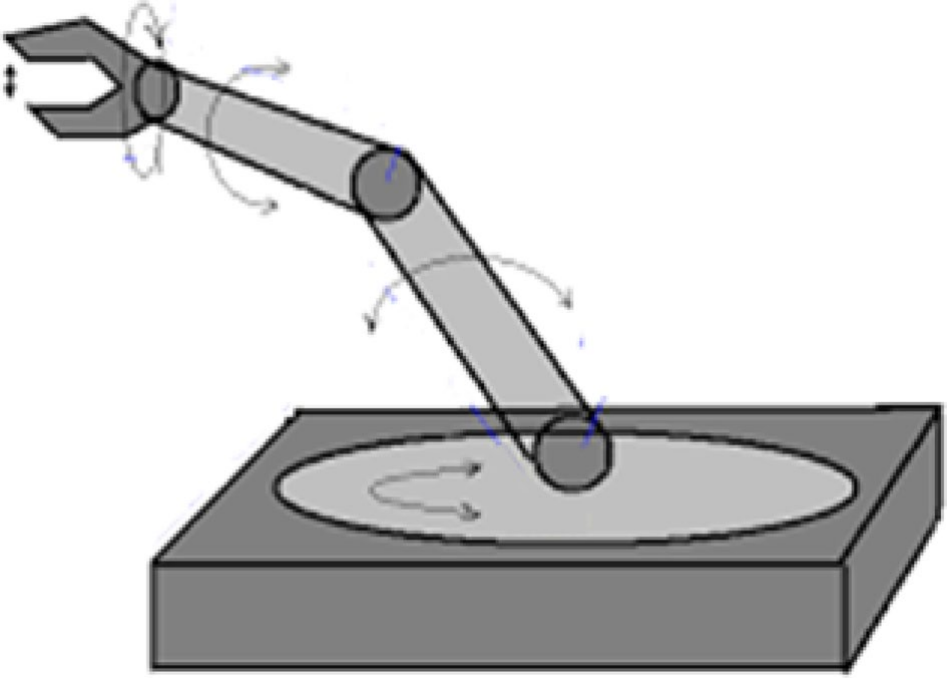
Robot arm.

It is usual for the Degree of Freedom (DOF) to express robotic structures and their arm’s functioning. The dynamic drive system of the robotic arm manipulator is the most essential and valuable element in all types of robotic structures and depends on the factors such as rise time, friction, setup time and motion of joints to be emulated. These parameters must be correctly adjusted for accurate motions with any conventional or modern approach. The setup time plays a vital part in all elements, as it talks about various transitory responses that must be taken into account throughout the robotic design. Due to their setup time, the robots are classified as low-speed and high-speed robots, with an increased set-up time for a low-speed robot. The time of adjustment also regulates the robot vibrations during operation. Joint motion or action is another important characteristic, which is primarily determined by the angular movement of the joints. It is shown in Fig. 1. In addition to motion, the action requires electricity as wToer to calculate these joint motions accurately, friction losses are eliminated, which can be efficiently utilized.

The dynamics model of a serious n-link rigid robot can be written as Eq. (1)^19^.

1 $$M\left(\dot{q}\right)\ddot{q}+C\left(q,\dot{q}\right)+g\left(q\right)=\tau$$

### Robot dynamics parameter estimation using PSO

To ensure precise torque control of the robot, the PSO inertial and frictional parameters of robot dynamics must be correctly calculated. It may be carried out using traditional methods, but its precision is doubtful, thus extracting these features requires PSO since PSO is one of the finest population-based optimization algorithms to succeed over its rivals^20^. As one of the most efficient stochastic optimization methods, the PSO algorithm has been proven over many reported studies, including non-linear problems, non-differentiable problems as well as problems with high dimensions and control problems. Each valued solution space response is assessed with a fitness function to find the best solution. Particles may have accumulated cognitive and socializing. In the neural network, the weight matrix is entered as a particle array which is randomly initialized/updated using mathematical modeling.

2 $$w\left(t+1\right)=w\left(t\right)+\Delta w\left(t+1\right),$$

3 $$parent\Delta w\left(t+1\right)=w\left(t\right)+{c}_{1}. raparentparentnd\left(\right).\left[pBest \left(t\right)-w\left(t\right)\right]+ {c}_{2}. rand\left(\right). \left[gBest \left(t\right)- w\left(t\right)\right],$$

Where w, c 1, c 2 is continuous inertia, cognitive and social acceleration^21^.

**Figure 2:**
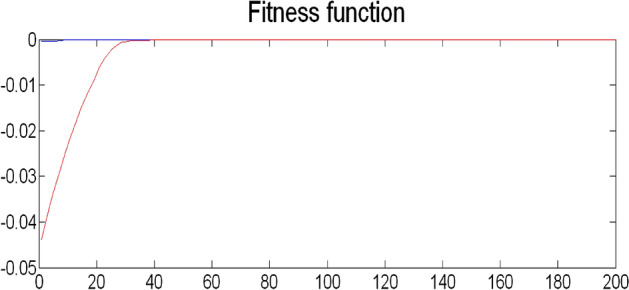
Fitness function to generate MF.

**Figure 3:**
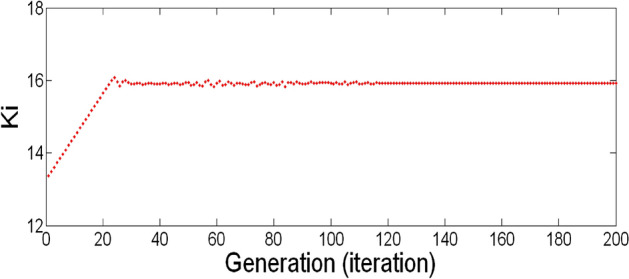
Exact ranges to generate K_i_ membership function.

**Figure 4:**
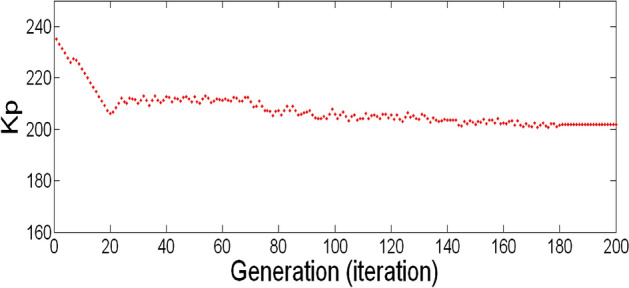
Exact ranges to generate the K_p_ membership function.

**Figure 5:**
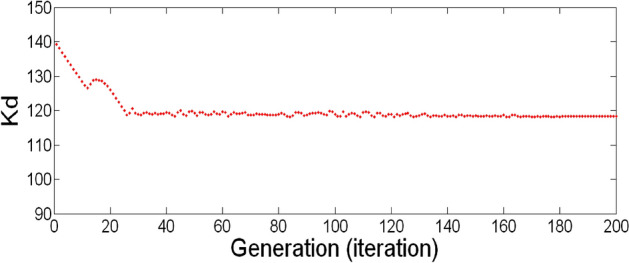
To produce the Kd membership function, the exact ranges.

**Figure 6:**
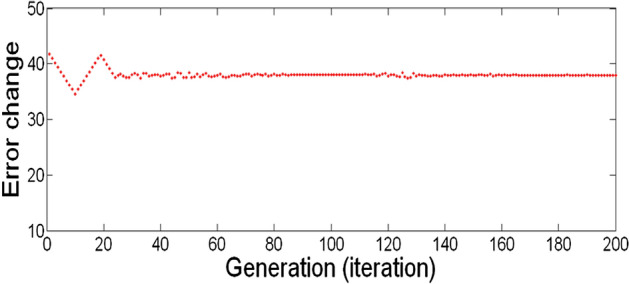
Exact ranges to generate change in the error membership function.

The best result of the particle is pBest. It tends to reflect its prior conduct. gBest is the best worldwide result of accurate particulate matter in the entire population so far. The highest speed Vmax is the key parameter linked to PSO. High speed helps to measure the resolution and to check the search area. The superior solution can be surpassed by particles when the value is extremely large and trapped in the optimal local value if the value is small^22–24^. The accurate fitness function is presented and shown in Fig. 2. Figure 3 explained the exact ranges to generate K_i_ membership function. Figure 4 illustrates the exact ranges to generate the K_p_ membership function. Figure 5 depicts to produce the K_d_ membership function. Figure 6 represented the exact ranges to generate change in the error membership function. The fitness function and the associated membership functions are depicted, which describing the Kp,Ki and Kd membership function and error changes of the input signal.

## Fuzzy controller

The fuzzy controller process is divided into four parts to get the desired result: fuzzification, rule basis, deduction, and defuzzification. PSO is utilized in the earlier part to build ranges for the extraction of membership features, which is essential for fluid processing. This foggy member logic turns a conventional input signal into a foggy signal^25^. The rule basis or knowledge base sets the rule, which is utilized to decide the inputs to a given task using the inference process. Finally, the de-fuzzing transforms the fuzzy output into a narrow output.

### Gaussian membership function

**Figure 7:**
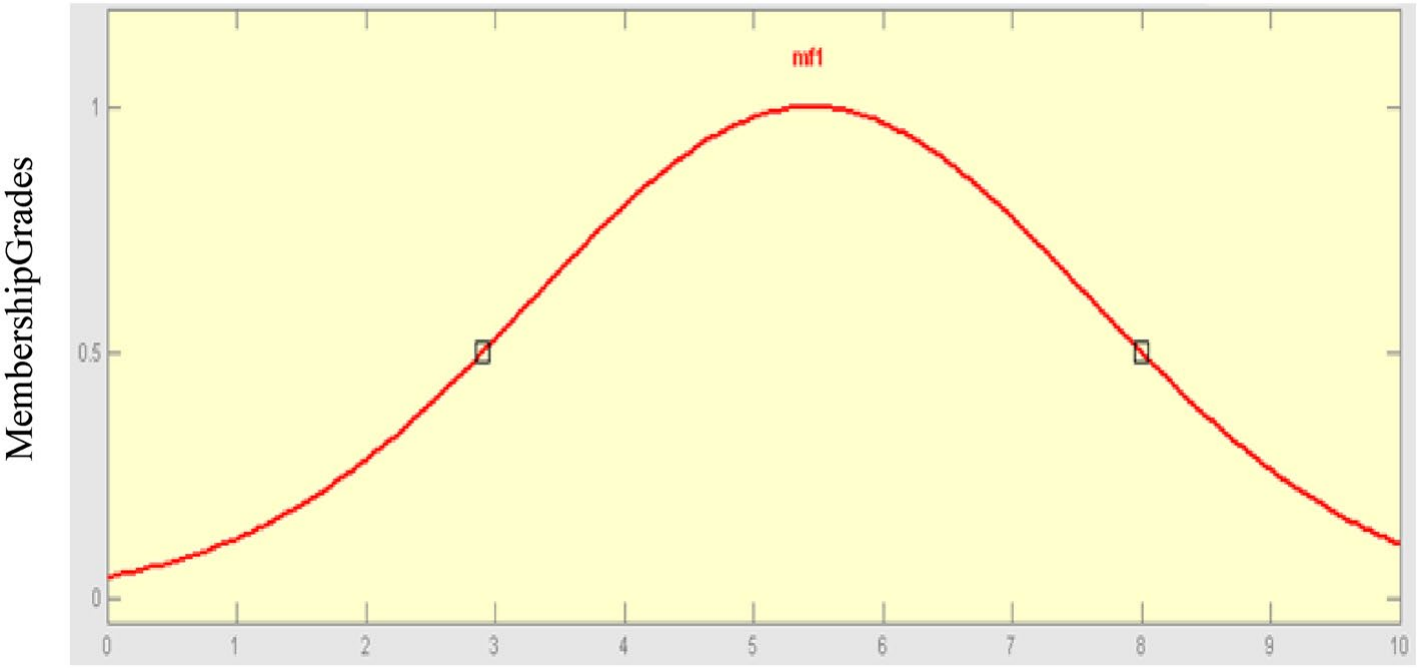
Gaussian membership function.

Equation (4) depicts the function of a Gaussian membership function, which is denoted by two parameters$, c,\sigma$. Figure 7 shows a plot of Gaussian membership.

4 $$gaussmf\left(x;c,\sigma \right)={e}^{\frac{-1}{2}{\left(\frac{x-c}{\sigma }\right)}^{2}}$$

c and identify a Gaussian membership function. Where c denotes the member's center and $\sigma$ denotes the member’s function breadth.

### Sigmoidal method membership function

**Figure 8:**
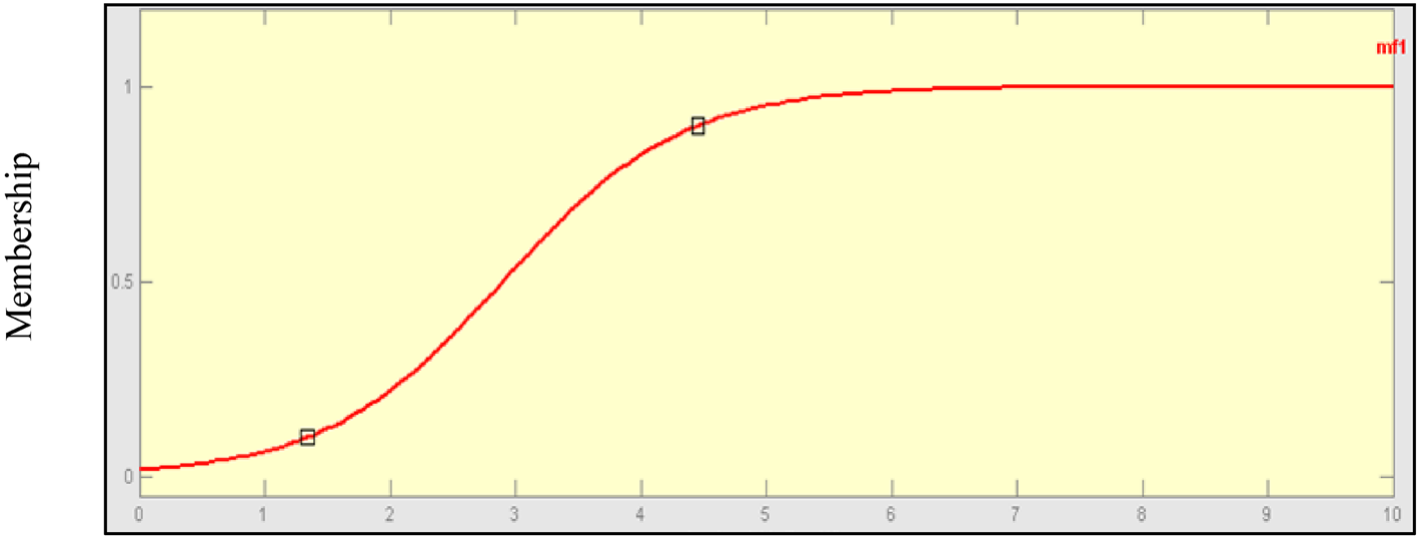
Sigmoidal membership function.

A sigmoid method membership function is distinct by the equation as given below and its plot is shown in Fig. 8.

5 $$f\left(x;a,c\right)=\frac{1}{1+exp[-a\left(x-a\right)]}$$

The fuzzy controller’s sigmoidal method membership function is essentially open to the left or right depending on the position of the input parameter. Sigmoidal membership hence functions perfectly for representing notions such as highly negative ones. For example, the unique sigmoidal function of sigma may be demonstrated.

### Defuzzification methods

Defuzzification implies that the crisp value derives from a flushed collection a representative value. There are several defusing strategies. Some of the most used methods such as the centroid, the sum center, and the maximum mean are discussed here. The Centroid technique is also called the Gravity Method Center and obtains the z* center in which discourse Z is set A. the centroid technique is specified by the terms;

6 $${z}^{*}= \frac{{\int }_{z}{\mu }_{A}(z)zdz}{{\int }_{z}{\mu }_{A}(z)dz}$$

**Figure 9:**
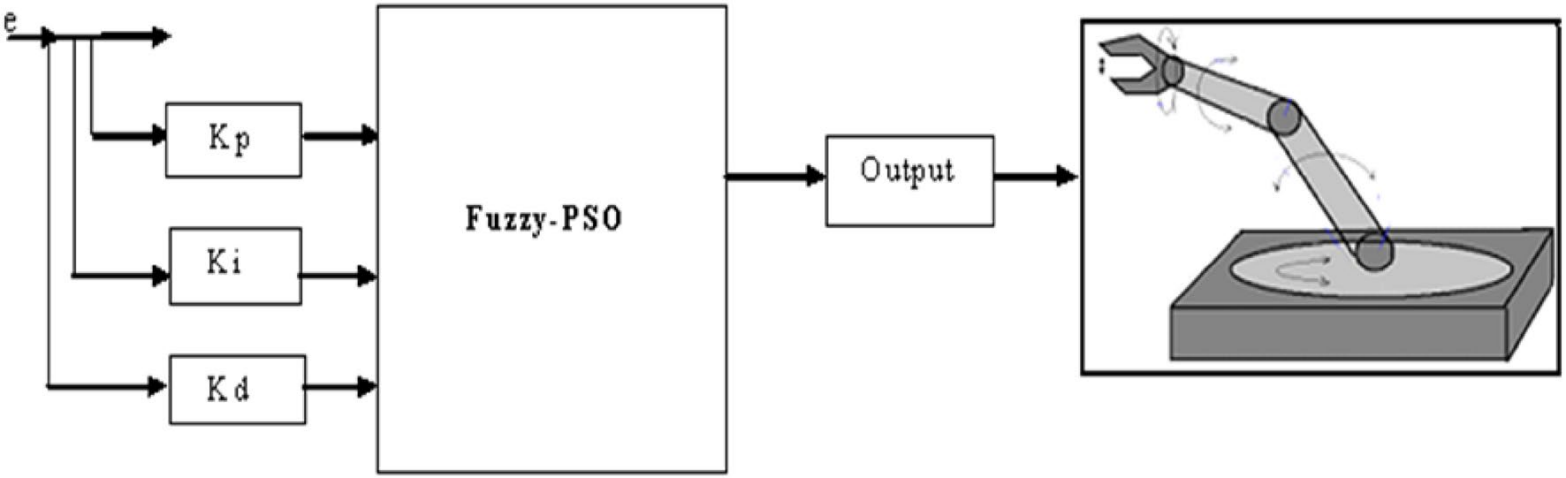
Structure of controller for robot arm.

The continuous function of fuzzy membership, where uA(z) is the aggregated MF output, which is the most often used defuzzification technique. The estimated values of the probability distribution function are comparable to the computation. In this study, the fugitive control is mostly focused on trying to deliver a non-linear action via fugitive reasoning for the controller's output. In this case, the controller's total gain is changed based on the fuzzy inference system and the continued progress became correct. The design process entails changing the outputs about inputs, as the PID controller for the regulation of robotic action is being examined in particular by the arm motions as illustrated in Fig. 9.

The first stage is to define the input and outputs of the Fuzzy logic. In the first stage, the system’s-controlled outputs consist of two inputs and three outputs, e(t) error change, and Kp, Ki, and Kd. Next, the fuzzification technique is performed with the major sets of fuzzy value and membership functions of both systems inputs and outputs. Mamdani inference mechanism is used to identify relationships between input and output^26,27^.

## Fuzzy supervisory control

### Estimation of the gain of K_i_, K_d_ and Kponerror e(t) and change of error increment $\Delta$ e(t)

**Figure 10:**
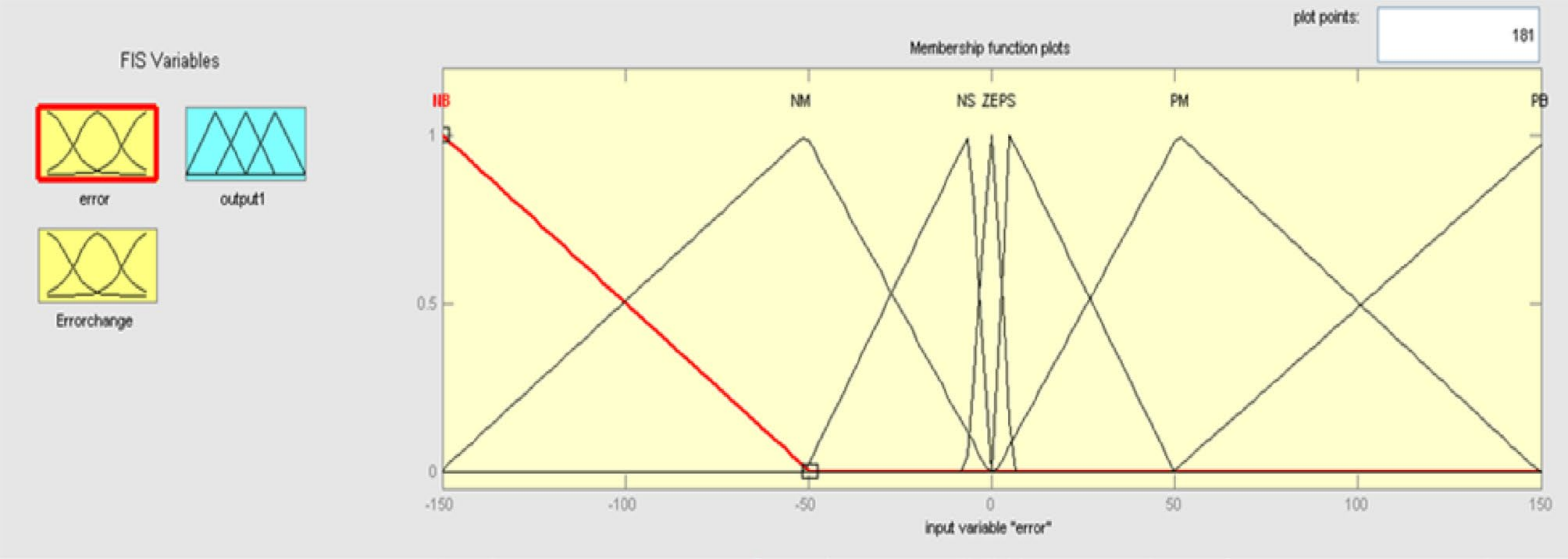
Membership function for input error.

**Figure 11:**
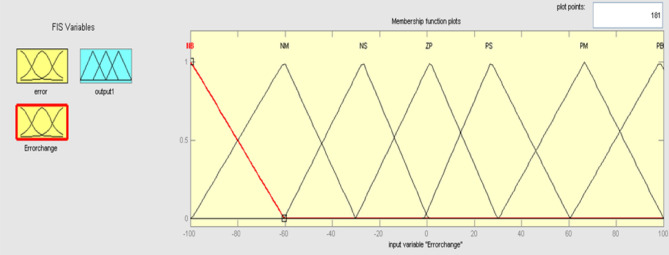
Membership function for input change in error.

The Fuzzy logic control is a loop system consisting of a mix of fluid and PID. The PID regulator yield reaction is generally altered by the fizzy controller and for all three parameters the control logic is performed using fuzzy e(t) and change of error ∆e(t). The error change are the fluke control error parameter, including e(t) with a range [− 150,150] and [− 100,100] with oste(t), is discovered on an experimental basis as shown in Fig. 10, and the error change membership in Fig. 11.

**Figure12:**
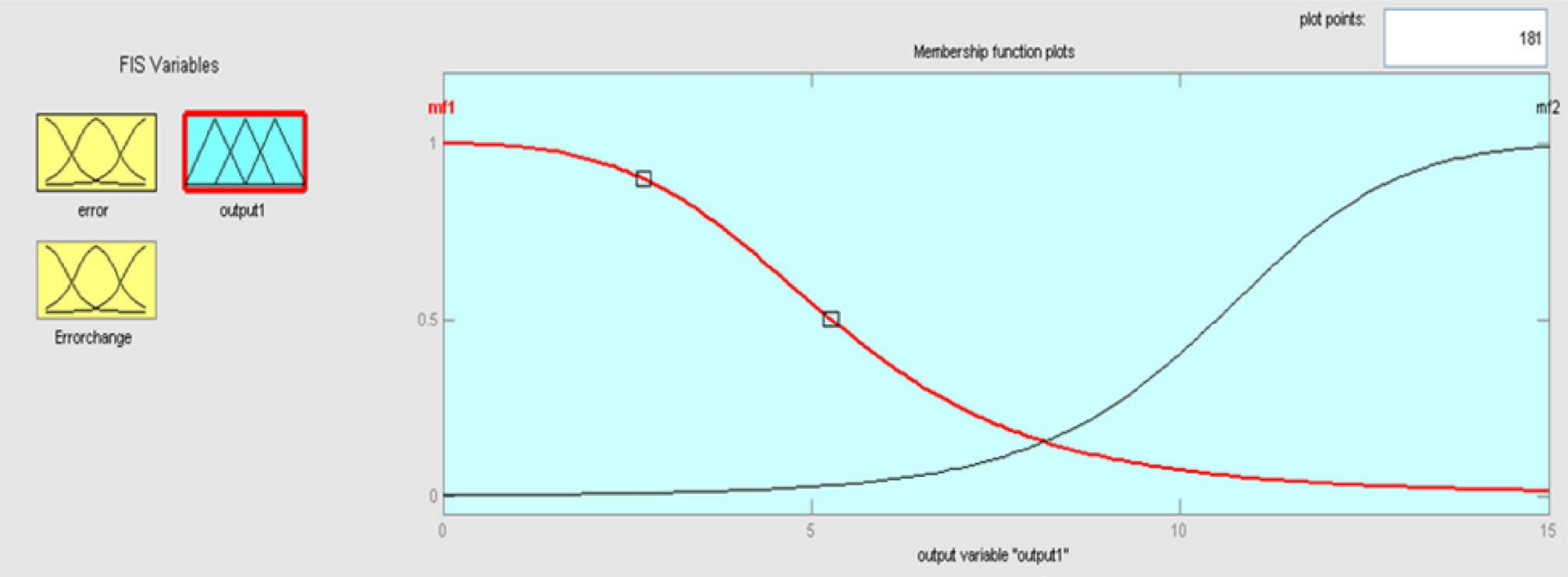
Membership function for output Kp.

**Figure 13:**
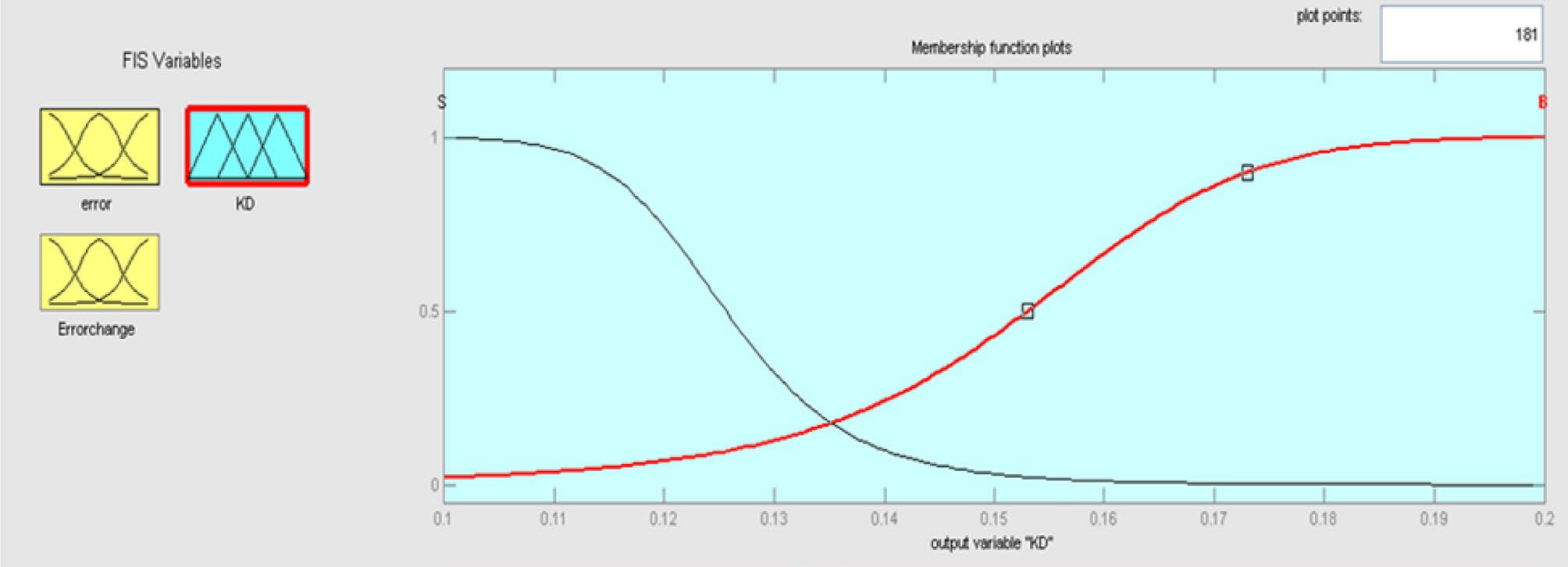
Membership function for output Kd.

**Figure 14:**
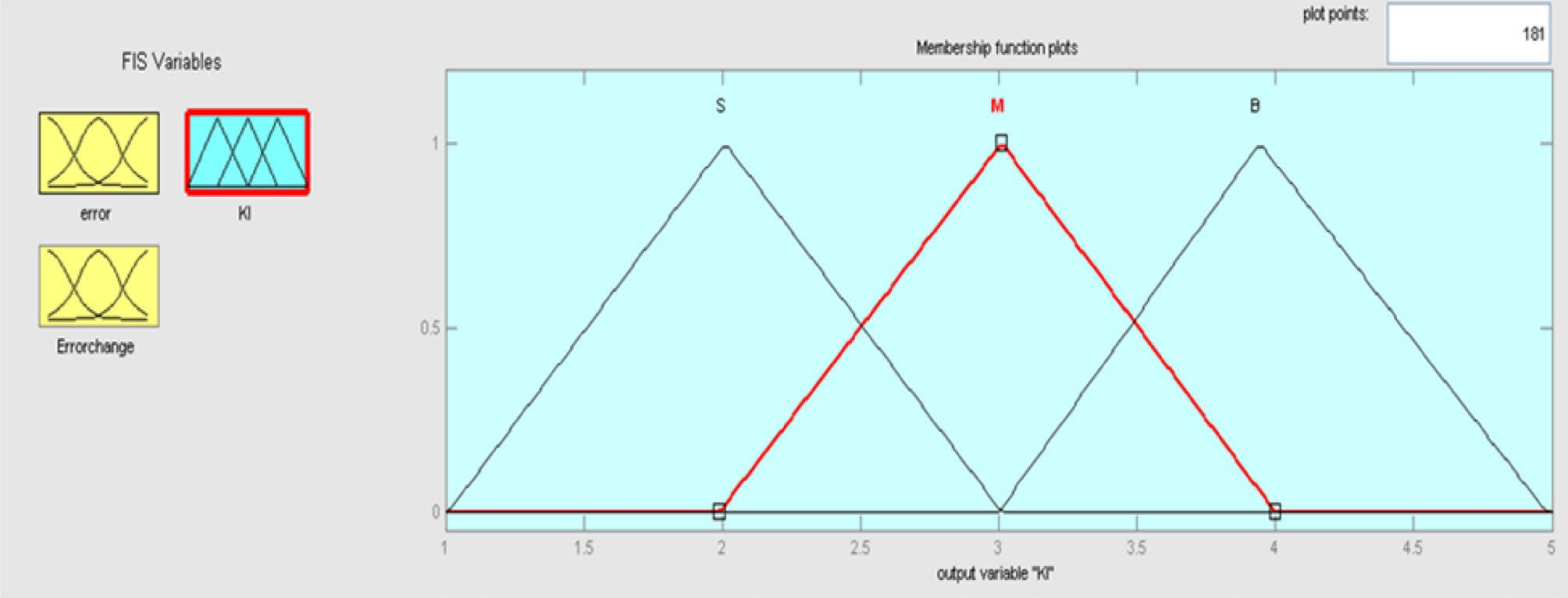
Membership function for output Ki.

Kp, Ki, and Kd are the output variables for the fuzzy control, as previously mentioned, which employ triangular, gaussian, and signals. KP and Kd have two levels of fuzzy member functions in the sigmoidal form as shown in Figs. 12 and 13, and Ki has 3 membership phases as shown in Fig. 14.

The language values for the fuzzy rules are KP and Kd as Minor (M) and Large (L), while the linguistic values for Ki are Minor (M), Intermediate (I), and Large (L). As mentioned before, these linguistic values are set by PSO.

There are few effective samples for training PSO at the beginning of the control and tracking signal transition. Due to its difficulty in convergent, the PSO identifier is feed forward. Control system errors are recorded, and poor effects are observed. Inverse models for control systems cannot be accurately established by the PSO identifier at this time^28^. At this time, the PSO controller output also has a high level of uncertainty. Therefore, we need to suppress the output of the PSO controller in order to reduce the impact of this uncertainty on the system. Fuzzy inference produces smaller output values as control error increases. The magnetic levitation ball system changes radically when the tracking signal changes violently^29,30^. To adjust to the change in control system state, the inverse model of PSO cannot converge quickly. Currently, there has been a large increase in error change rate, which necessitates weakening the PSO control function, that is, lowering fuzzy inference output. As a result, fuzzy inference should produce a smaller output value when the error change rate is larger. Increased sampling increases the accuracy of the PSO inverse model, improves the feedforward control effect of the PSO controller, and reduces errors and error change rates. In this situation, fuzzy inference output values should be increased and PSO controller output adjustments should be reduced. Thus, a fuzzy inference should produce a smaller output when error e and error change rate ec are smaller than one another.

### Estimation of KP and Ki for Torque control

**Figure 15:**
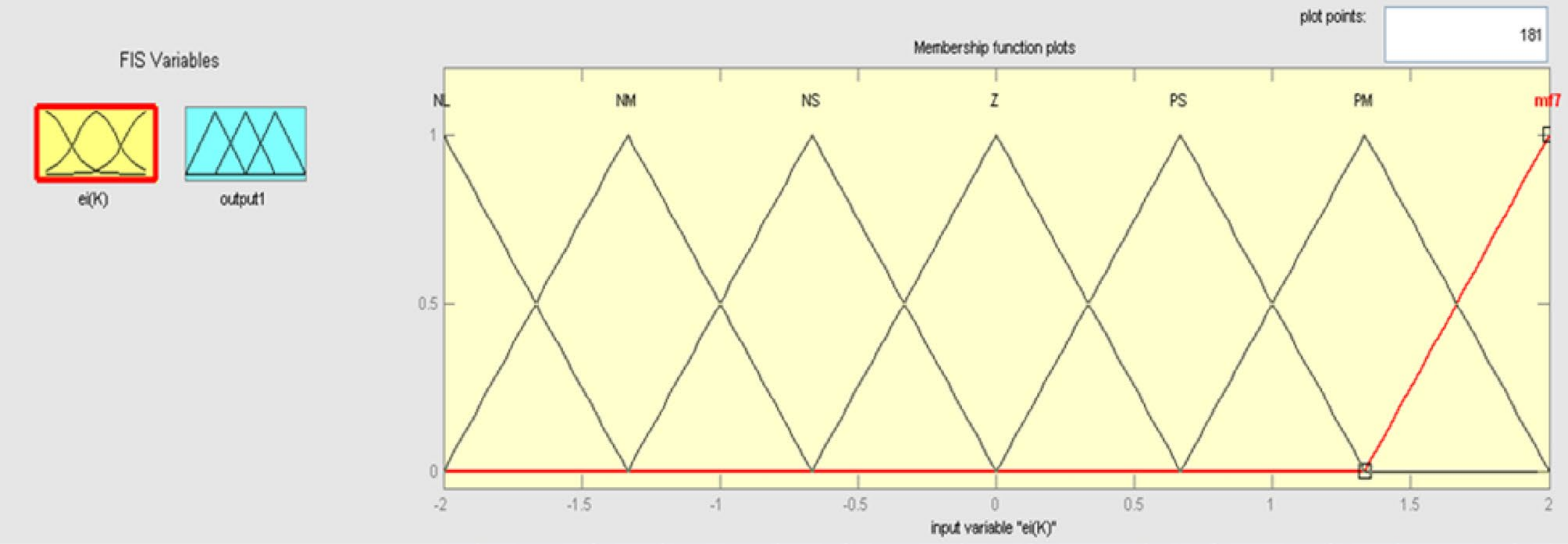
Membership function forinput *ei(k)*.

The robot's engine and torque are controlled by its current. The torque is thought to be regulated by the current mistake ei(k) and the current error *cei*(k) at each sampling time. Figure 15 shows the Membership function for input *ei(k).*

**Figure 16:**
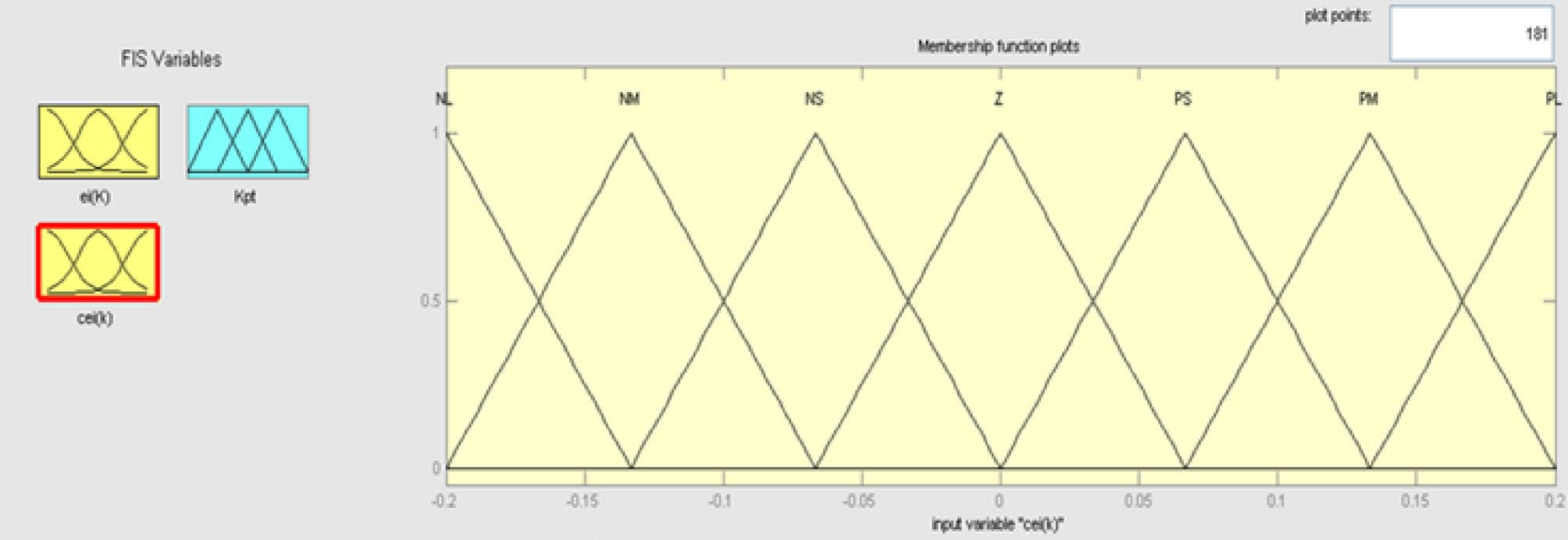
Membership function for input *cei(k)*.

Initially, while approaching the benchmark from a standing condition, the motor engine requires an increased Kp value and must have a reduced Ki value to prevent over-shooting. That is why Ki and Kp’s reaction to device outputs was calculated with classic [30, 50] and [0.5, 1.5] technologies. The suggested fuzzy logic control continually adapts the existing location with integral gain and proportional gain coefficients shows in Fig. 16.

**Figure 17:**
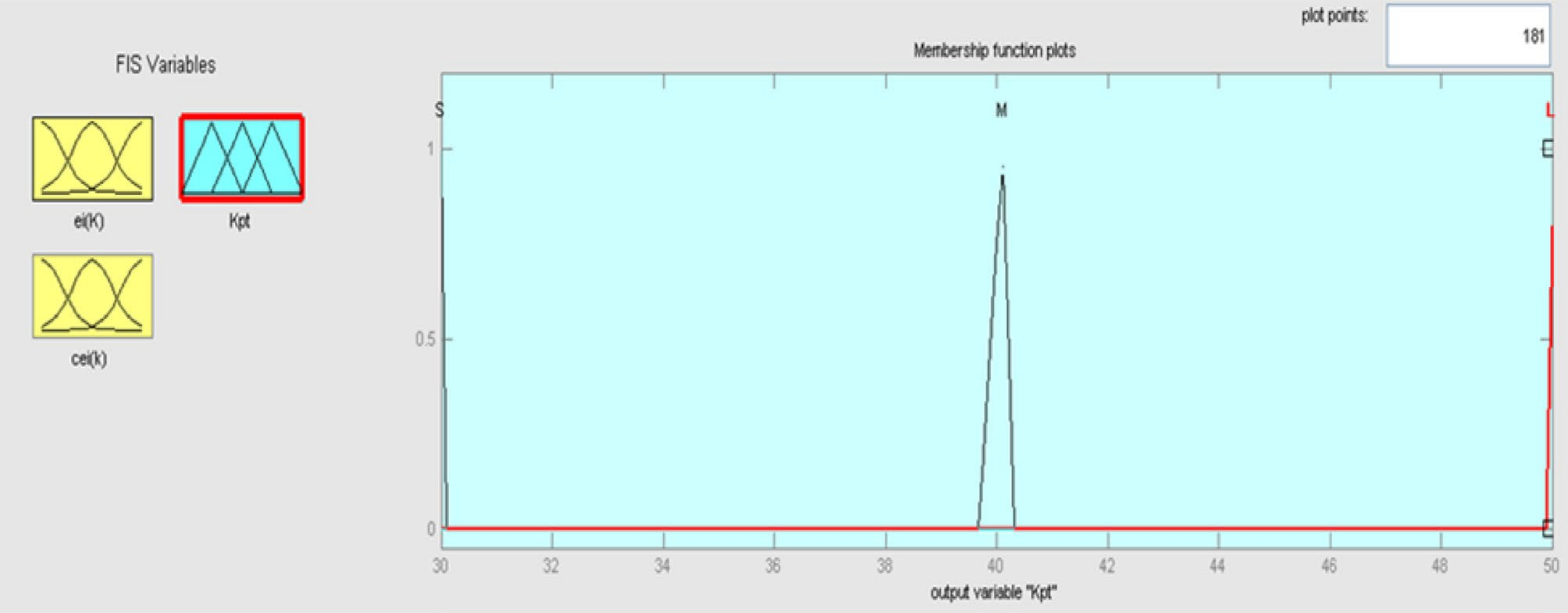
Membership function for output Kp.

**Figure 18:**
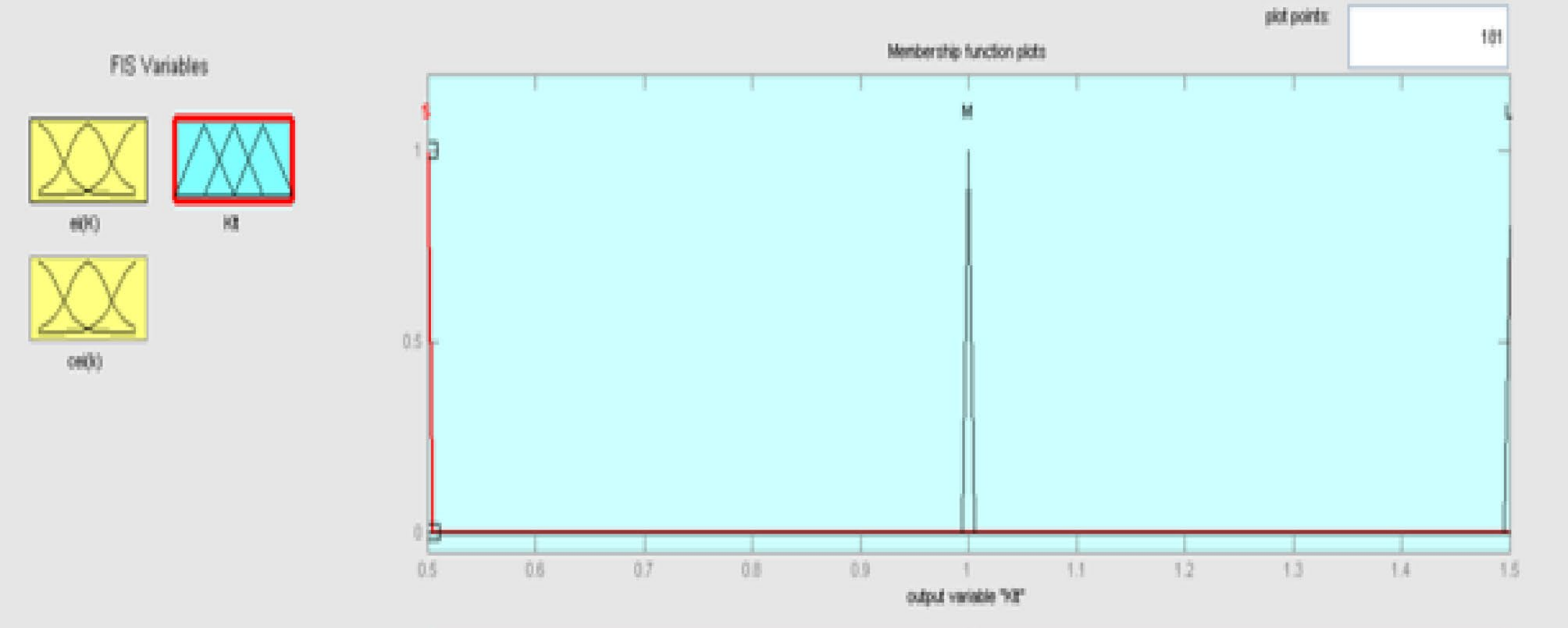
Membership function for output Ki.

The Fuzzy controller has two condition outputs to determine the optimal values of the proportional and integral gain of Kp and Ki coefficients. Minor (M), Intermediate (I), and Large (L) are language definitions of fuzzy logic output variables (L). Figures 17 and 18 demonstrate the membership functions for the outputs Kp and Ki.

## Robotic materials

The material commonly used in the construction of robots is steel. Some robotic systems use metal and structural composites of carbon fiber and other materials to help them function in clean rooms, for the space program, or other "high tech" applications. Strength requirements and operating environment influence material selection. There are many types of metals and composites available on the market today. Materials are selected in a very detailed manner. Robot bases and robot arms are commonly constructed from steel, cast iron, and aluminum, although other materials can also be used. The softer nature of aluminum makes it easier to work with. The strength of steel, however, is several times greater. Aluminium was the material of choice for the parts. Selection has not been direct. Shoulders have the option of carrying more weight than other parts. For the most part, the robotic arm is made out of aluminum 6061 alloys that are used for the majority of its parts. A precipitation-hardened aluminum alloy known as 6061, which contains magnesium and silicon as its key alloying elements, is classified as a precipitation-hardened aluminum alloy. This material possesses good mechanical properties, is a good welder, is widely used for extrusion, and is second in popularity only to 6063 in terms of popularity. Among the most common alloys of aluminum, it is used in general-purpose applications. Depending on the temperature at which a material is heated, its properties are greatly affected. 69 GPa (10,000 ksi) is Young's modulus. There is no maximum tensile strength for 6061 annealed sheet metal (6061-O temper) higher than 120 MPa (18,000 psi), and there is no maximum yield strength higher than 55 MPa (8000 psi) for 6061 annealed sheet metal. In addition, the material has an elongation (the length it will stretch before it fails) between 25 and 30% before it fails.The end effector is also made of ABS, which is lighter than aluminum 6061. ABS is used instead of Aluminum 6061 because it reduces the load on the end effector. Low-cost engineering thermoplastic ABS (acrylonitrile–butadiene–styrene) can be machined, fabricated, and thermoformed easily. Thermoplastic material is highly resistant to chemical agents, stress, and creep. As a result, ABS offers good impact resistance, heat resistance, chemical resistance, abrasion resistance, tensile strength, surface hardness, rigidity, as well as electrical properties.

## Result and discussion

**Figure 19:**
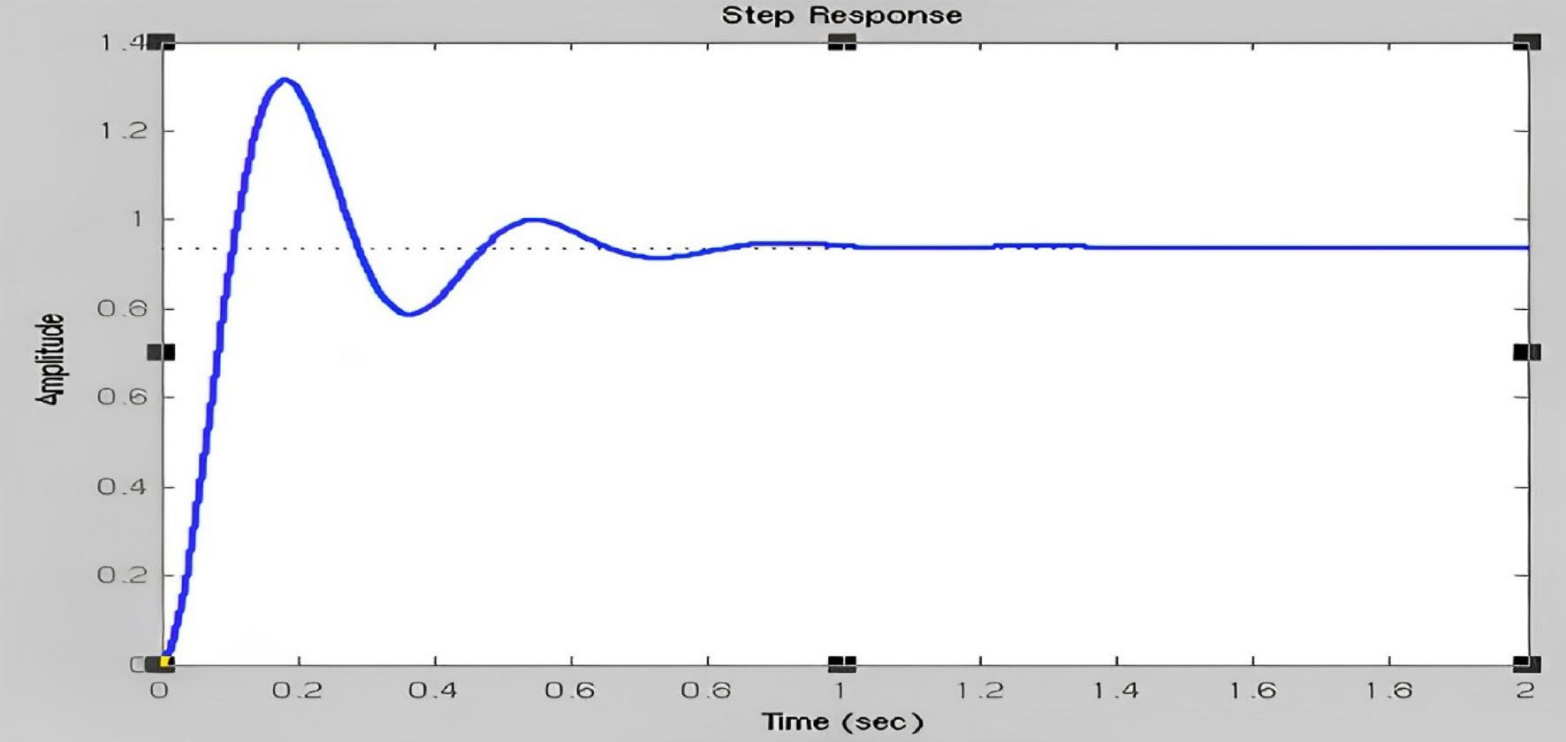
Improved response of rise time & steady-state error.

**Figure 20:**
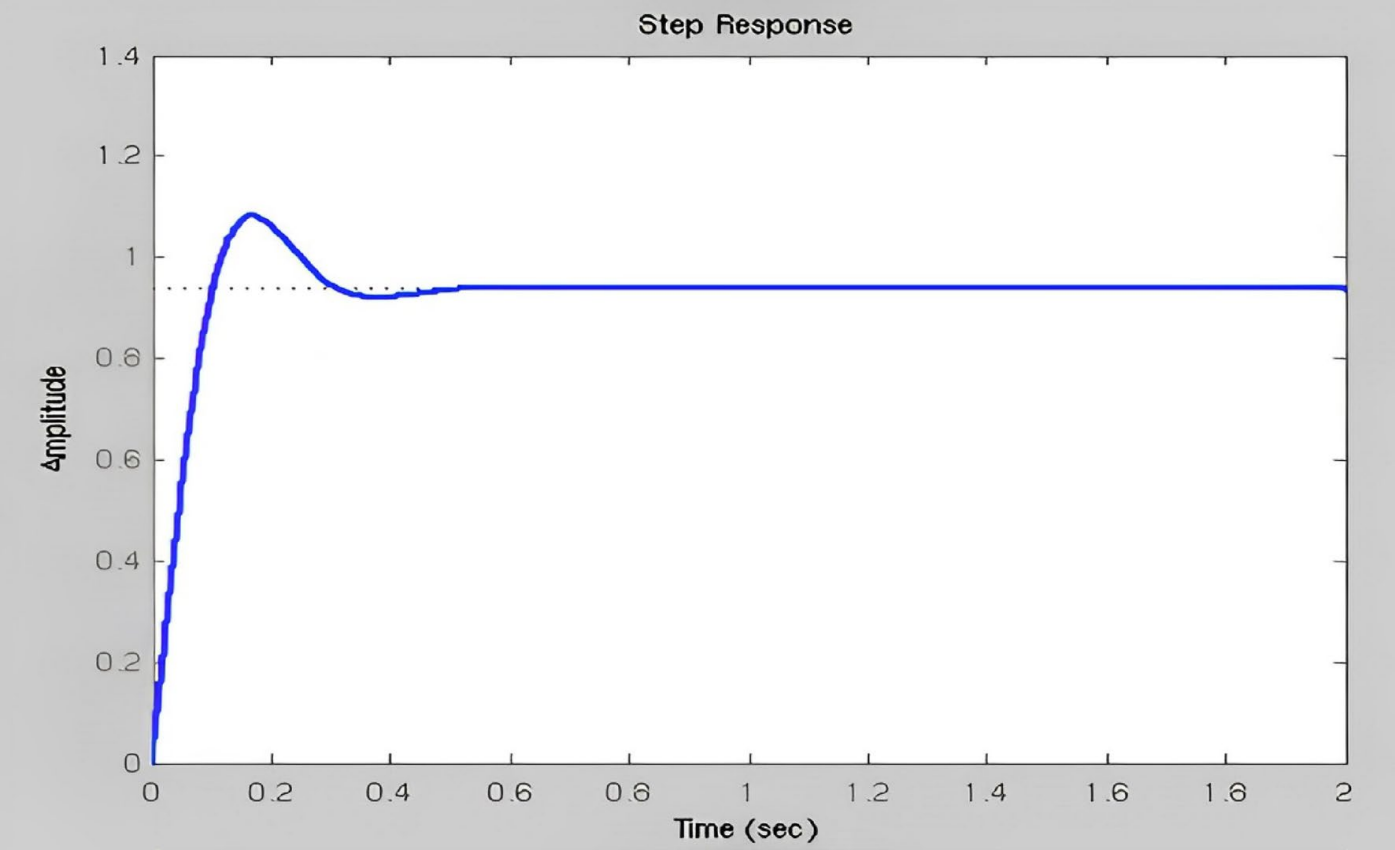
Minimized overshoot.

**Figure 21:**
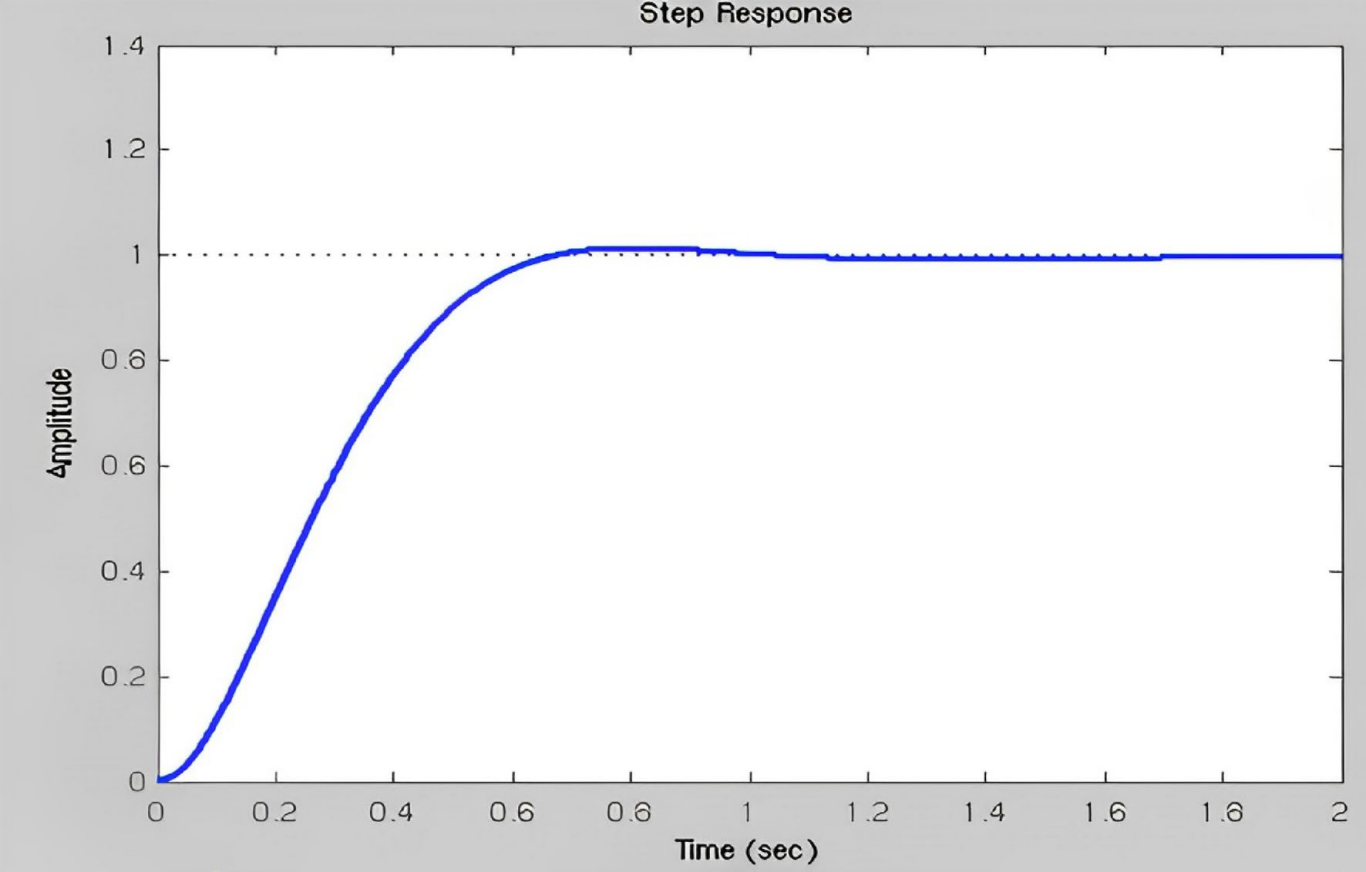
Disregarded steady-state error for over-Shoot.

The fuzzy output is immediately provided as a DC servomotor input to perform mechanical operations by selecting approximately the transfer function (TF) of the DC servo motor. Kp, Ki, and Kd play important roles as previously indicated. Conventional Time to rise: It has been determined that the values of the input step size were 10 and 90 percent higher, the total time necessary to shift a signal from a lower to a higher value. The maximum overflow is usually described as the ratio between the largest response curve estimates and the intended frame response, often computed based on step inputs. The Overshoot percentage is the ratio between the input size of the maximum value and the step size in continues. Thesteady-state error refers to the change in the predicted ultimate output, and the real response is seen when the system is in a steady state and its behavior is estimated to continue when the system is continuous. These answers are based on their gain from Figs. 19, 20 and 21. This illustrates how the gain time is improved based on the fuzzy logic simulated gain and an improved response to the rise time based on the steady-state mistake in Fig. 19.

PI and PID controllers group the proportional, integral, and derivative controller components. The Ziegler Nichols approach is one of the relevant frameworks for this task. By the Ziegler Nichols approach the Kd and Ki are adjusted first and only the proportional control is activated. The estimate of Kp should be continuously increased until the framework shows non-stop deviations. The Kp estimate is now indicated as the Kp limit with the T-limit for the fluctuation period, after which derivative and integral controller gain values are generated using fuzzy logic approaches. Once the regulators have achieved their gains, the sequential response of the fluid-based logic controller is transferred to the DC servo motor. In some special circumstances, the best reaction in certain conditions might go against the provided values in the table and can potentially lead to large swings.

**Figure 22:**
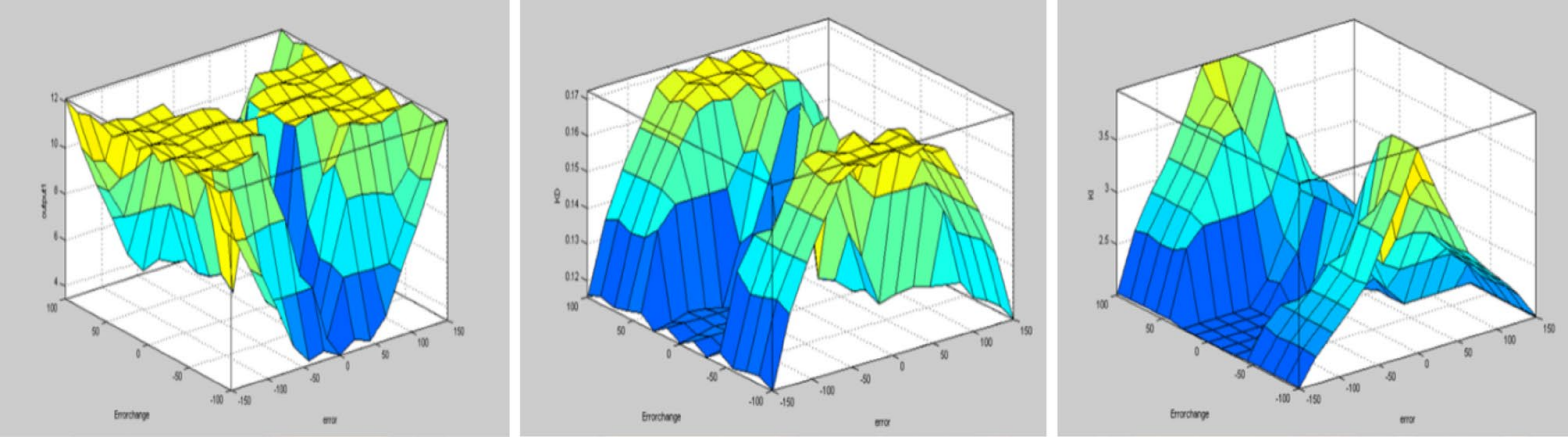
Surface view for Kp, Kd and Ki.

**Figure 23:**
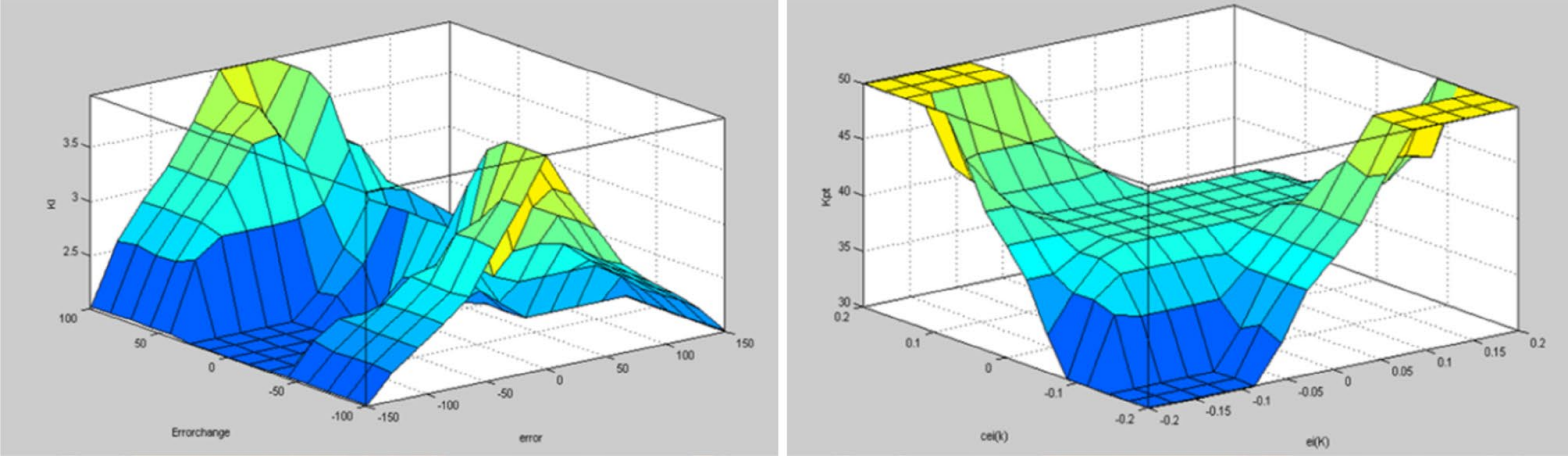
Surface view for Kp, and Ki for torque control.

In such cases, the gain of the computed values should be “intuitively” adjusted to get superior solutions. The findings displayed in Fig. 22 are achieved by using fluid rules. The reason that the concerted outputs for PID gain are generated is thatKp and Ki have very excellent torque control of the engine surface view and have a proper climb, peak overshoot, and steady-state error. Figure 23 shows the Surface view for Kp, and Ki for torque control.

The preceding parts cover the development of inputs and outcomes. To use a specified arm, the fuzzy surveillance controller must modify its parameter gain to provide a rapid climb, a smaller overflow, and a no steady condition error,which are displayed in Table 1. To do this, every gain parameter should be shown with enough e(t) and daily ranges (t). There may be opportunities for mistakes in positive and negative regions. To change the parameter in the positive area, there must be a greater integral gain, more proportional gain, and less derivative gain. To eliminate and minimize the error entirely from any system, the integral gain must be calibrated and the derivative gain adjusted to accelerate the system.

**Table 1:** Comparison of Robot Characteristics with Conventional controller, Fuzzy controller, Fuzzy-PSO controller.

Controller	Robot characteristics		
	Maximum peak overshoot in percentage (Mp%)	Rise time of signal (Tr)	Steady-state error
Conventional controller	–	0.098	0.064
Fuzzy controller	0.04	0.086	0.036
Fuzzy-PSO controller	0.08	0.060	0.007

## Conclusion

This article, therefore, utilizes the possibility of superlatively controlling the robotic arm actuator by the hybrid modeling of Fuzzy logic with the PSO to manage the DC linear servo engine. Simulated results using the traditional technique are good for linear changes while Fuzzy’s findings are well-suitable for the operation and control of the robot for both structured and unstructured features. Fuzzy logic performance precision is improved by support for cognitive and social behaviors of fitness particles by using partial swarm optimization and the derivation of the ranges of structures for each rule of the fuzzy membership function. It is done particularly to extract the characteristics. The results of the simulation with Fuzzy Logic-PSO have been significantly improved and provide results in less than one cycle compared to previous techniques. In an industrialized manufacturing environment, these industrial robots are extremely useful. This type of arm is typically used for materials handling, painting, and welding, among other applications. Robotic technologies will undoubtedly alter the way things are done in the industries in which they are being implemented. Robotics is being used in various industries by entrepreneurs with similar perspectives. Industries such as manufacturing, pharmaceuticals, FMCG, packaging, and inspection are primarily impacted by robotics. It is also possible to see a bit of robotics in the technologies. Education and defense are also promising sectors. The world has recently experienced a PC revolution and a mobile revolution. Now, it is time for the inevitable robotics revolution. A significant increase in amateur robotic enthusiasts, as well as open-source robotic tools and platforms, and the investment of global players such as Google, FESTO, and Tesla in robotics, indicates that significant development in this field will take place within the next five to ten years.

## Data Availability

The datasets generated and/or analyzed during the current study are not publicly available. But are available from the corresponding author on reasonable request.
